# Overcoming Model Uncertainty — How Equivalence Tests Can Benefit From Model Averaging

**DOI:** 10.1002/sim.10309

**Published:** 2025-03-19

**Authors:** Niklas Hagemann, Kathrin Möllenhoff

**Affiliations:** ^1^ Institute of Medical Statistics and Computational Biology (IMSB), Faculty of Medicine University of Cologne Cologne Germany

**Keywords:** bootstrap, dose–response models, gene expression, model averaging, model‐based equivalence tests, time‐response models

## Abstract

A common problem in numerous research areas, particularly in clinical trials, is to test whether the effect of an explanatory variable on an outcome variable is equivalent across different groups. In practice, these tests are frequently used to compare the effect between patient groups, for example, based on gender, age, or treatments. Equivalence is usually assessed by testing whether the difference between the groups does not exceed a pre‐specified equivalence threshold. Classical approaches are based on testing the equivalence of single quantities, for example, the mean, the area under the curve or other values of interest. However, when differences depending on a particular covariate are observed, these approaches can turn out to be not very accurate. Instead, whole regression curves over the entire covariate range, describing for instance the time window or a dose range, are considered and tests are based on a suitable distance measure of two such curves, as, for example, the maximum absolute distance between them. In this regard, a key assumption is that the true underlying regression models are known, which is rarely the case in practice. However, misspecification can lead to severe problems as inflated type I errors or, on the other hand, conservative test procedures. In this paper, we propose a solution to this problem by introducing a flexible extension of such an equivalence test using model averaging in order to overcome this assumption and making the test applicable under model uncertainty. Precisely, we introduce model averaging based on smooth Bayesian information criterion weights and we propose a testing procedure which makes use of the duality between confidence intervals and hypothesis testing. We demonstrate the validity of our approach by means of a simulation study and illustrate its practical relevance considering a time‐response case study with toxicological gene expression data.

## 1 Introduction

In numerous research areas, particularly in clinical trials [1, 2], a common problem is to test whether the effect of an explanatory variable on an outcome variable is equivalent across different groups. Equivalence is usually assessed by testing whether the difference between the groups does not exceed a pre‐specified equivalence threshold. The choice of this threshold is crucial as it resembles the maximal amount of deviation for which equivalence can still be concluded. One usually chooses the threshold based on prior knowledge, as a percentile of the range of the outcome variable or resulting from regulatory guidelines. Equivalence tests provide a flexible tool for plenty of research questions. For instance, they can be used to test for equivalence across patient groups, for example, based on gender or age, or between treatments. Moreover, they are a key ingredient of bioequivalence studies [3, 4], investigating whether two formulations of a drug have nearly the same effect and are hence considered to be interchangeable.

Classical approaches [5, 6] are based on testing the equivalence of single quantities, for example, the mean, the area under the curve (AUC) or other values of interest. However, when differences depending on a particular covariate are observed, these approaches can turn out to be not very accurate. Instead, considering the entire covariate range, describing for instance the time window or a dose range, has recently been proposed by testing equivalence of whole regression curves. Such tests [7, 8, 9] are typically based on the principle of confidence interval inclusion. However, a more direct approach applying various distance measures has been introduced by Dette et al. [10] which turned out to be particularly more powerful. Based on this, many further developments [11, 12, 13, 14], for example, for different outcome distributions, specific model structures and/or responses of higher dimensions, have been introduced.

All these approaches have one thing in common: they base on the assumption that the true underlying regression model is known. In practice this usually implies that the models need to be chosen manually, either based on prior knowledge or visually. Hence, these approaches [15, 16] might not be robust with regard to model misspecification and, consequently, suffer from problems like inflated type I errors or reduced power. One idea to tackle this problem is implementing a testing procedure which explicitly incorporates the model uncertainty. This can be based on a formal model selection procedure, see, for example, Möllenhoff et al. [17] who propose conducting a classical model choice procedure prior to preforming the equivalence test.

An alternative to this is the incorporation of a model averaging approach into the test procedure. As outlined by Bornkamp [18] model selection has some disadvantages compared to model averaging. Particularly, model selection is not stable in the sense that minor changes in the data can lead to major changes in the results [19]. This also implies that model selection is non‐robust with regard to outliers. In addition, the estimation of the distribution of post model selection parameter estimators is usually biased [20, 21]. Model averaging is omnipresent whenever model uncertainty is present, which is, besides other applications, often the case in parametric dose response analysis. Besides practical applications, there are also several methodological studies regarding model averaging in dose–response studies [22, 23, 24] and Bornkamp et al. [25] incorporated model averaging as an alternative to model selection in their widely used dose‐finding method MCPMod.

Therefore, in this paper, we propose an approach utilizing model averaging rather than model selection. There are frequentist as well as Bayesian model averaging approaches. The former almost always use the smooth weights structure introduced by Buckland et al. [26] These weights depend on the values of an information criterion of the fitted models. Predominantly, the Akaike information criterion (AIC) [27] is used but other information criteria can be used as well. While only few of the Bayesian approaches perform fully Bayesian inference (see, e.g., Ley and Steel [28]), the majority makes use of the fact that the posterior model probabilities can be approximated by weights based on the Bayesian information criterion (BIC) [29] that have the same smooth weights structure as the frequentist weights [30]. Despite the prevalence of the AIC and BIC, other information criteria are sometimes used as well: Price et al. [31] suggested to use the deviance information criterion (DIC) [32], which is the Bayesian analog to the AIC. Hence, it bases on the samples of a Markov chain Monte Carlo simulation rather than on the log‐likelihood. Hjort and Claeskens [33] introduced model averaging based on the focused information criterion (FIC) [34]. In contrast to other information criteria, the FIC does not aim for the best overall fit but focuses directly on a parameter of primary interest (e.g., the mean, the median or a specific quantile). Therefore, it favors models which lead to the best estimated precision with regard to this focus parameter. Occasionally, model averaging also bases on cross‐validation, for example, jackknife model averaging [35], or machine learning methods, for example, random forests or boosting [36]. Alternatively, rather simple model averaging approaches with fixed model weights exist as well, for example, using equal weights. However, the performance of such approaches strongly depends on prior knowledge and can easily lead to (partial) model missspecification. For a more general introduction to model averaging techniques the reader is referred to, for example, Fletcher [37] or Claeskens and Hjort [38] and an overview specifically focusing on dose–response models is given by Schorning et al. [22]

In this paper, we propose an equivalence test incorporating model‐averaging and hence overcoming the problems caused by model uncertainty. Precisely, we first make use of the duality between confidence intervals and hypothesis testing and propose a test based on the derivation of a confidence interval. By doing so, we both guarantee numerical stability of the procedure and provide confidence intervals for the measure of interest.

We demonstrate the usefulness of our method with the example of toxicological gene expression data. In this application, using model averaging enables us to analyze the equivalence of time–response curves between two groups for 1000 genes of interest without the necessity of specifying all 2000 correct models separately, thus avoiding both a time‐consuming model selection step and potential model misspecifications.

The paper is structured as follows: In Section 2, dose–response models and the concept of model averaging are succinctly discussed. In Section 3, the testing approach is introduced, proposing three different variations. Finite sample properties in terms of Type I and II error rates are studied in Section 4. Section 5 illustrates the method using the toxicological gene expression example before Section 6 closes with a discussion.

## 2 Model Averaging for Dose–Response Models

### 2.1 Dose–Response Models

We consider two different groups, indicated by an index l=1,2, with corresponding response variables $y_{\mathrm{lij}}$ with Y⊆R denoting the set of all possible outcomes. There are $i=1,\ldots ,I_{l}$ dose levels and $j=1,\ldots ,n_{\mathrm{li}}$ denotes the observation index within each dose level. For each group the total number of observations is $n_{l}$ and n is the overall number of observations, that is, $n_{l}=\sum_{i=1}^{I_{l}}n_{\mathrm{li}}$ and $n=n_{1}+n_{2}$. For each group we introduce a flexible dose–response model

$$y_{\mathrm{lij}}=m_{l}\left(x_{\mathrm{li}},\theta_{l}\right)+e_{\mathrm{lij}},\;j=1,\ldots ,n_{\mathrm{li}},\;i=1,\ldots ,I_{l},\;l=1,2,$$

where $x_{\mathrm{li}}\in X\subseteq R$ is the dose level, that is, the deterministic explanatory variable. We assume the error terms $e_{\mathrm{lij}}$ to be independent, have expectation zero and finite variance $\sigma_{l}^{2}$. The function $m_{l}\left(\cdot \right)$ models the effect of $x_{\mathrm{li}}$ on $y_{\mathrm{lij}}$ via a regression curve with $\theta_{l}\in R^{\dim\left(\theta_{l}\right)}$ being its parameter vector. We assume $m_{l}\left(\cdot \right)$ to be twice continuously differentiable. In dose–response studies, as well as in time‐response studies, often either a linear model

(1) $$m_{l}\left(x,\theta_{l}\right)=\beta_{l0}+\beta_{l1}x$$

a quadratic model

(2) $$m_{l}\left(x,\theta_{l}\right)=\beta_{l0}+\beta_{l1}x+\beta_{l2}x^{2}$$

an emax model

(3) $$m_{l}\left(x,\theta_{l}\right)=\beta_{l0}+\beta_{l1}\frac{x}{\beta_{l2}+x}$$

an exponential (exp) model

(4) $$m_{l}\left(x,\theta_{l}\right)=\beta_{l0}+\beta_{l1}\left(\exp\left(\frac{x}{\beta_{l2}}\right)-1\right)$$

a sigmoid emax (sigEmax) model

(5) $$m_{l}\left(x,\theta_{l}\right)=\beta_{l0}+\beta_{l1}\frac{x^{\beta_{l3}}}{{\left(\beta_{l2}\right)}^{\beta_{l3}}+x^{\beta_{l3}}}$$

also known as Hill model or 4pLL‐model, or a beta model

(6) $$m_{l}\left(x,\theta_{l}\right)=\beta_{l0}+\beta_{l1}\left(\frac{{\left(\beta_{l2}+\beta_{l3}\right)}^{\beta_{l2}+\beta_{l3}}}{{\left(\beta_{l2}\right)}^{\beta_{l2}}+{\left(\beta_{l3}\right)}^{\beta_{l3}}}\right){\left(\frac{x}{s}\right)}^{\beta_{l2}}{\left(1-\frac{x}{s}\right)}^{\beta_{l3}}$$

where s is a fixed scaling parameter, is deployed [39, 40, 41, 42]. These models strongly vary in the assumed underlying dose–response relation, for example, in terms of monotonicity, and consequently in the shape of their curves. Therefore, choosing a suitable dose–response model is crucial for all subsequent analyses.

However, in practical applications the true underlying model shape is in general unknown. Thus, it might not always be clear which functional form of ((1), (2), (3), (4), (5), (6)) should be imployed. A possible answer to this is implementing model averaging which, as outlined in Section 1, has several advantages over the simpler alternative of model selection.

### 2.2 Model Averaging

As outlined before, frequentist as well as Bayesian model averaging approaches usually both use the same smooth weights structure introduced by Buckland et al. [26] and Wasserman [30], respectively. Accordingly, by leaving out the group index l=1,2 for better readability the averaged model is given by

(7) $$m\left(x,\hat{\theta }\right)≔\sum_{k=1}^{K}w_{k}\;m_{k}\left(x,{\hat{\theta }}_{k}\right)$$

where the $m_{k}\left(x,{\hat{\theta }}_{k}\right),k=1,..,K,$ correspond to the K candidate models,

(8) $$w_{k}=\frac{\exp(-0.5I\left(m_{k}\left(x,{\hat{\theta }}_{k}\right)\right)}{\sum_{\tilde{k}=1}^{K}\exp\left(-0.5I\left(m_{\tilde{k}}\left(x,{\hat{\theta }}_{\tilde{k}}\right)\right)\right)}$$

are the corresponding weights and I(⋅) is an information criterion with smaller values corresponding to better model fit. All information criteria considered here are based on the calculation of a penalized log‐likelihood for each candidate model. Usually the AIC is used for frequentist model averaging, while the BIC is usually deployed for Bayesian model averaging [22]. A notable special case arises when the number of parameters is the same for all candidate models: the smooth AIC and smooth BIC weights are exactly equal in this situation, as the penalty term vanishes from Equation (8). In this case the weights only depend on the value of the respective log‐likelihood.

### 2.3 Inference

As the parameter estimation is conducted for each of the candidate models separately, it is not influenced by the subsequent model averaging. Therefore, here the index k is left out. Inference can be based on an ordinary least squares (OLS) estimator, that is, minimizing

$$\sum_{i=1}^{I_{l}}\sum_{j=1}^{n_{\mathrm{li}}}{\left(y_{\mathrm{lij}}-m_{l}\left(x_{\mathrm{li}},\theta_{l}\right)\right)}^{2},\;l=1,2$$

In general, no distributional assumption is needed regarding the error terms, they just need to be independent, have expectation zero and finite variance $\sigma_{l}^{2}$ as outlined in Section 2.1. However, by making a distributional assumption, a maximum likelihood estimator can also be deployed. Usually, normality of the error terms, that is

$$e_{\mathrm{lij}}\overset{\mathrm{iid}}{∼}N\left(0,\sigma_{l}^{2}\right),\;l=1,2$$

with log‐likelihood

(9) $$\ell \left(\theta_{l},\sigma_{l}^{2}\right)=-\frac{n_{l}}{2}\ln\left(2\pi \sigma_{l}^{2}\right)-\frac{1}{2\sigma_{l}^{2}}\sum_{i=1}^{I_{l}}\sum_{j=1}^{n_{\mathrm{li}}}{\left(y_{\mathrm{lij}}-m_{l}\left(x_{\mathrm{li}},\theta_{l}\right)\right)}^{2},\;l=1,2$$

is assumed but other distributions can be considered as well. Under normality both approaches are identical and, hence, lead to the same parameter estimates ${\hat{\theta }}_{l}$. From (9) a maximum likelihood estimator for the variance

(10) $${\hat{\sigma }}_{l}^{2}=\frac{1}{n_{l}}\sum_{i=1}^{I_{l}}\sum_{j=1}^{n_{\mathrm{li}}}{\left(y_{\mathrm{lij}}-m_{l}\left(x_{\mathrm{li}},{\hat{\theta }}_{l}\right)\right)}^{2},\;l=1,2$$

can be derived as well. In R inference is performed with the function fitMod from the package DoseFinding [25, 41] which performs OLS estimation. The value of the log‐likelihood needed for the AIC or BIC is then calculated by plugging the OLS estimator into the log‐likelihood (9).

Even though smooth AIC and smooth BIC weights share the same structure, there are differences regarding their asymptotic properties. As outlined by several authors [33, 38, 43], the asymptotic distribution of model average estimators is in general no longer a normal distribution due to being a non‐linear transformation of normal distributions. An exception to this is model averaging with fixed weights: due to the weights being non‐random, the asymptotic distribution of the model average estimator is a linear combination of normal distributions and, hence, also a normal distribution.

Wang et al. [43] investigated asymptotic properties for smooth AIC and smooth BIC weights explicitly. Under regulatory assumptions, the asymptotic distribution of the model average estimator using smooth AIC weights is in general non‐normal. In contrast, using smooth BIC weights leads to the asymptotic distribution of the model average estimator being a normal distribution. With regard to our study, it will turn out that this is an important advantage of smooth BIC weights, as asymptotic normality is used in order to show the asymptotic validity of the testing approach introduced in Section 3. In addition, the BIC‐based weights provide additional interpretability due to being approximately equal to the posterior model probabilities.

Comparing the different information criteria, we can summarize that smooth BIC weights are advantageous in terms of their ability to be integrated into the framework of model‐based equivalence testing. For smooth BIC weights, there is sufficient asymptotic theory to justify the asymptotic validity of the test. In contrast, smooth AIC weights do not provide the necessary asymptotic properties to guarantee a theoretical justification. Smooth DIC weights are not applicable because frequentist inference is performed. Smooth FIC weights are problematic because there is no focus parameter and one searches for the overall best fitting curves. Asymptotic theory is not (yet) available for most cross‐validation or machine learning methods. Finally, fixed weights depend on prior knowledge and can lead to (partial) misspecification of the model. Therefore, we will use smooth BIC weights for the rest of this paper.

## 3 Model‐Based Equivalence Tests Under Model Uncertainty

### 3.1 Equivalence Testing Based on Confidence Intervals

Model‐based equivalence tests [10, 44] have been introduced in terms of the $L^{2}$‐distance, the $L^{1}$‐distance or the maximal absolute deviation (also called $L^{\infty }$‐distance) of the model curves. Although all of these approaches have their specific advantages and disadvantages as well as specific applications, subsequent research [11, 12, 14, 17] is predominately based on the maximal absolute deviation due to its easy interpretability. Accordingly, we state the hypotheses

(11) $$H_{0}:d\geq \varepsilon \;\mathrm{vs}.\;H_{1}:d<\varepsilon$$

of equivalence of regression curves with respect to the maximal absolute deviation, that is

$$d=\max_{x\in X}|m_{1}\left(x,\theta_{1}\right)-m_{2}\left(x,\theta_{2}\right)|$$

is the maximal absolute deviation of the curves and ε is the pre‐specified equivalence threshold, meaning that a difference of ε is believed not to be clinically relevant. The test statistic is given as the estimated maximal deviation between the curves

(12) $$\hat{d}=\max_{x\in X}|m_{1}\left(x,{\hat{\theta }}_{1}\right)-m_{2}\left(x,{\hat{\theta }}_{2}\right)|.$$

As the distribution of $\hat{d}$ under the null hypothesis is in general unknown, it is usually either approximated based on a parametric bootstrap procedure or by asymptotic theory. In Dette et al. [10], the asymptotic validity of both approaches is proven, but the corresponding simulation study shows that the bootstrap test outperforms the asymptotic test in finite samples. For the bootstrap test several studies [10, 11, 17] show reasonable results for finite samples across applications.

However, in light of practical application, this approach can have two disadvantages: First, it does not directly provide confidence intervals (CI) which provide useful information about the precision of the test statistic. Further, they would have an important interpretation analogously to their interpretation in classical equivalence testing known as TOST [5] (two one‐sided tests), where the bounds of the confidence interval are typically compared to the confidence region of [−ε,ε].

Second, it requires the estimation of the models under the constraint of being on the edge of the null hypothesis, that is, the maximal absolute deviation being equal to ε (see Algorithm 1 in Dette et al. [10]). Technically, this is usually conducted using augmented Lagrangian optimisation. However, with increasing model complexity, this becomes numerically challenging. In the context of model averaging, these numerical issues are particularly relevant since all models would need to be estimated jointly as they need to jointly fulfill the constraint. This leads to a potentially high dimensional optimisation problem with a large number of parameters. In addition, for model averaging the side constraint has a highly complex structure because with every parameter update not only the model curves change but also the model weights do.

ALGORITHM 1

1. Obtain parameter estimates ${\hat{\theta }}_{\mathrm{lk}},k=1,\ldots ,K_{l},l=1,2,$ for the candidate models, either via OLS or maximum likelihood optimisation (see Section 2.3). Determine the averaged models from the candidate models using Equation (7), that is, by calculating
$$m_{l}\left(x,{\hat{\theta }}_{l}\right)=\sum_{k=1}^{K_{l}}w_{\mathrm{lk}}\;m_{\mathrm{lk}}\left(x,{\hat{\theta }}_{\mathrm{lk}}\right),\;l=1,2,$$
  with weights (8) as well as the variance estimator ${\hat{\sigma }}_{l}^{2},l=1,2$ from Equation (10). Alternatively, use fixed weights instead of weighting scheme (8).
2. Calculate the test statistic (12).
3. Execute the following steps:
  - Obtain bootstrap samples by generating data according to the model parameters ${\hat{\theta }}_{l}=\left({\hat{\theta }}_{l1},\ldots ,{\hat{\theta }}_{\mathrm{lK}}\right),l=1,2,$ and the weights $w_{l1},\ldots ,w_{\mathrm{lK}},l=1,2,$ obtained in step 1. Under the assumption of normality, that is
$$y_{\mathrm{lij}}^{*}∼N\left({\hat{\mu }}_{\mathrm{li}},{\hat{\sigma }}_{l}^{2}\right),\;j=1,\ldots ,n_{\mathrm{li}},i=1,\ldots ,I_{l},l=1,2$$
    where
$${\hat{\mu }}_{\mathrm{li}}=m_{l}\left(x_{\mathrm{li}},{\hat{\theta }}_{l}\right)=\sum_{k=1}^{K_{l}}w_{\mathrm{lk}}\;m_{\mathrm{lk}}\left(x_{\mathrm{li}},{\hat{\theta }}_{\mathrm{lk}}\right),\;i=1,\ldots ,I_{l},l=1,2$$
    Alternative distributions with corresponding mean and variance can be used as well.
  - From the bootstrap samples, estimate the models $m_{l}\left(x_{\mathrm{li}},{\hat{\theta }}_{l}^{*}\right),l=1,2$ as in step (1) and the test statistic
(15) $${\hat{d}}^{*}=\max_{x\in X}|m_{1}\left(x,{\hat{\theta }}_{1}^{*}\right)-m_{2}\left(x,{\hat{\theta }}_{2}^{*}\right)|.$$
  - Repeat steps (3.a) and (3.b) $n_{\text{boot}}$ times to generate replicates ${\hat{d}}_{1}^{*},\ldots ,{\hat{d}}_{n_{\text{boot}}}^{*}$ of ${\hat{d}}^{*}$. Let ${\hat{d}}_{\left(1\right)}^{*}\leq \ldots \leq {\hat{d}}_{\left(n_{\text{boot}}\right)}^{*}$ denote the corresponding order statistic.
4. Calculate the CI using one of the following approaches:
  - *Percentile CI*: Obtain the estimated right bound of the percentile bootstrap CI as the (1−α) ‐quantile of the bootstrap sample
$$\hat{u}={\hat{q}}^{*}\left(1-\alpha \right)={\hat{d}}_{\left(\left\lfloor n_{\text{boot}}\left(1-\alpha \right)\right\rfloor \right)}^{*}.$$
  - *Hybrid CI*: Obtain the estimator for the standard error of $\hat{d}$ as $\hat{\mathrm{se}}\left(\hat{d}\right)=\sqrt{\hat{\mathrm{Var}}\left({\hat{d}}_{1}^{*},\ldots ,{\hat{d}}_{n_{\text{boot}}}^{*}\right)}$ and the estimated right bound of the hybrid CI as
$$\hat{u}=\hat{d}+\hat{\mathrm{se}}\left(\hat{d}\right)z$$
5. Reject the null hypothesis in (11) and assess equivalence if
$$\varepsilon >\hat{u}$$

As an alternative to approximating the distribution under the null hypothesis, we propose to test hypotheses (11) based on the well‐known duality between confidence intervals and hypothesis testing [45]. This testing approach is similar to what Bastian et al. [44] introduced for the $L^{1}$‐distance of regression models. Therefore, let (−∞,u] be a one‐sided lower (1−α)‐CI for d which we can rewrite as [0,u] due to the non‐negativity of d, that is

P(d≤u)=P(d∈(−∞,u])=P(d∈[0,u])≥1−α

According to the duality between CI and hypothesis testing, we reject the null hypothesis and conclude equivalence if

(13) ε>u

This testing procedure is an α‐level test as

$$\;\;{\mathbb{P}}_{H_{0}}\left(\varepsilon >u\right)\leq P\left(d>u\right)=1-P\left(d\leq u\right)\leq 1-\left(1-\alpha \right)=\alpha$$

However, as the distribution of $\hat{d}$ is in general unknown, obtaining u is again a challenging problem. It is obvious from (13) that the quality of the testing procedure crucially depends on the quality of the estimator for u. If the CI is too wide the test procedure is conservative and lacks power. In contrast, a too narrow CI can lead to type I error inflation due to not reaching the desired coverage probability 1−α. We propose three different possibilities to calculate the CI, namely

1. CI based on a parametric percentile bootstrap,
2. asymptotic CI based on the asymptotic distribution of $\hat{d}$ derived by Dette et al. [10], and
3. a hybrid approach using the asymptotic normality of $\hat{d}$ but estimating its standard error based on a parametric bootstrap.

One‐sided CIs based on a parametric percentile bootstrap can be constructed in the same way Möllenhoff et al. [17] proposed for two‐sided CIs. In order to do so, they obtain parameter estimates (either via OLS or maximum likelihood optimisation), generate bootstrap data from these estimates and calculate the percentiles from the ordered bootstrap sample. The resulting test is similar to what Bastian et al. [44] derived for the $L^{1}$‐distance of regression models. That is

$$\left[0,{\hat{q}}^{*}\left(1-\alpha \right)\right],$$

where ${\hat{q}}^{*}\left(1-\alpha \right)$ denotes the (1−α)‐quantile of the ordered bootstrap sample.

Asymptotic CIs can be derived directly from test (5.4) of Dette et al. [10] and are given by

(14) $$\left[0,\hat{d}+\sqrt{\frac{\hat{\mathrm{Var}}\left(d\right)}{n}}z\right]$$

where z is the (1−α)‐quantile of the standard normal distribution and $\hat{\mathrm{Var}}\left(d\right)$ is the closed‐form estimator for the variance of d given by equation (4.7) of Dette et al. [10] However, the asymptotic validity of this variance estimator is only given under the assumption that within X there is only one unique value $x_{0}$ where the absolute difference curve attains its maximum, that is, $x_{0}=\text{argma}x_{x\in X}|m_{1}\left(x,\theta_{1}\right)-m_{2}\left(x,\theta_{2}\right)|$ and, moreover, that this value $x_{0}$ is known. This does not hold in general as Dette et al. [10] give two explicit counterexamples in terms of two shifted emax or exponential models. In addition, in practical applications $x_{0}$ is in general not known and needs to be estimated. If the absolute deviation along x is small, the estimation of $x_{0}$ can become unstable leading to an unstable variance estimator. Moreover, as mentioned before, the simulation study of Dette et al. [10] shows that for finite samples the bootstrap test is superior to the asymptotic test.

Given the disadvantages of the asymptotic CI and especially of the underlying variance estimator, we introduce a hybrid approach which is a combination of both approaches. It is based on the asymptotic normality of $\hat{d}$ but estimates the standard error of $\hat{d}$ based on a parametric bootstrap leading to

$$\left[0,\hat{d}+\hat{\mathrm{se}}\left(\hat{d}\right)z\right]$$

where the estimator $\hat{\mathrm{se}}\left(\hat{d}\right)$ of the standard error of $\hat{d}$ is the empirical standard deviation of the bootstrap sample.

Under the assumptions introduced by Dette et al. [10] all three approaches are asymptotically valid. For the test based on the asymptotic CI, this follows directly from Dette et al. [10] This also applies to the hybrid CI‐based test due to $\hat{\mathrm{se}}\left(\hat{d}\right)$ being an asymptotically unbiased estimator for the standard error of $\hat{d}$ as outlined by Efron and Tibshirani [46]. The asymptotic validity of the percentile approach follows from Dette et al. [10] (Appendix: proof of Theorem 4). The finite sample properties of the three methods are compared in Section 4.1.

### 3.2 Model‐Based Equivalence Tests Incorporating Model Averaging

We now combine the model averaging approach presented in Section 2.2 with the CI‐based test introduced in Section 3.1. For the asymptotic test, that is, estimating $m_{l}\left(x,{\hat{\theta }}_{l}\right),l=1,2$ using Equation (7) with model weights (8) and then calculating the test statistic (12). Subsequently, the asymptotic CI (14) can be determined using the closed form variance estimator given by Dette et al. [10]. Using this CI, the test decision is based on decision rule (13).

The testing procedure of the percentile and hybrid approach is shown in Algorithm 1, where the first two steps are essentially the same as for the asymptotic test. The percentile test is conducted by performing Algorithm step 4a, while conducting step 4b instead leads to the hybrid test. In the following we will refer to this as Algorithm 1a and Algorithm 1b, respectively.

The asymptotic validity discussed at the end of Section 3.1 only transfers to averaged models if the asymptotic distribution of the model average estimator is normal. As outlined in Section 2.3, this is given for smooth BIC weights as well as for fixed weights.

## 4 Finite Sample Properties

In the following we investigate the finite sample properties of the proposed tests by a simulation study. In order to ensure comparability, we reanalyze the simulation scenarios given by Dette et al. [10] The dose range is given by X=[0,4] and data is observed for dose levels x=0,1,2,3 and 4 with equal number of observations $n_{\mathrm{li}}=\frac{n_{l}}{5}$ for each dose level. All three simulation scenarios use the same three variance configurations $\left(\sigma_{1}^{2},\sigma_{2}^{2}\right)\in \left\{\left(0.25, 0.25\right),\left(0.25, 0.5\right),\left(0.5, 0.5\right)\right\}$ as well as the same four different sample sizes $\left(n_{1},n_{2}\right)\in \left\{\left(10,10\right),\left(10,20\right),\left(20,20\right),\left(50,50\right)\right\}$ and the same significance level of α=0.05.

In the first simulation scenario the equivalence of an emax model and an exponential model is investigated. The other two simulation scenarios consist of testing for equivalence of two shifted models, either both being emax models or both being exponential models. In contrast to the first scenario where the absolute deviation of the models is observed at one unique $x_{0}$, this is not the case for the latter two scenarios. Here, the deviation of both models is constant across the whole dose range X=[0,4], that is

$$|m_{1}\left(x,\theta_{1}\right)-m_{2}\left(x,\theta_{2}\right)|=d\forall x\in X$$

as $m_{1}$, $m_{2}$ are just shifted.

Hence, for these two scenarios the asymptotic test is not applicable as its close form variance estimator bases on the uniqueness of $x_{0}$. Therefore, only simulation Scenario 1 is used to compare the three CI‐based tests to each other as well as to the results observed by Dette et al. [10] Subsequently, all three scenarios are used to compare the performance of the test using model averaging to the one based on the correct specification of the models as well as under model misspecification.

### 4.1 Finite Sample Properties of Confidence Interval‐Based Equivalence Testing

Prior to the investigation of the effect of model averaging onto the finite sample properties, we first inspect the performance of the CI‐based testing approach, denoted by (13), itself. For the asymptotic test, the CI is defined by (14). For the percentile as well as the hybrid approach, the tests are conducted as explained in Section 3.1 which is formally defined by setting $K_{1}=K_{2}=1$ in Algorithm 1.

As outlined before, simulation scenario 1 of Dette et al. [10] is given by tesing for the equivalence of an emax model (3) with $\theta_{1}=\left(\beta_{10},\beta_{11},\beta_{12}\right)=\left(1,2,1\right)$ and an exponential model (4) with $\theta_{2}=\left(\beta_{20},\beta_{21},\beta_{22}\right)=\left(\beta_{20},2.2, 8\right)$. It consists of 60 sub‐scenarios resulting from the three different variance configurations each being combined with the four different sample sizes and five different choices of $\beta_{20}\in \left\{0.25, 0.5, 0.75, 1,1.5\right\}$, leading to the corresponding deviations of the regression curves being d∈{1.5, 1.25, 1,0.75, 0.5}. The test is conducted for ε=1 such that the first three deviations are under the null hypothesis and, therefore, used to investigate the type I error rates. The latter two deviations correspond to the alternative and are used to estimate the power of the tests.

**FIGURE 1:**
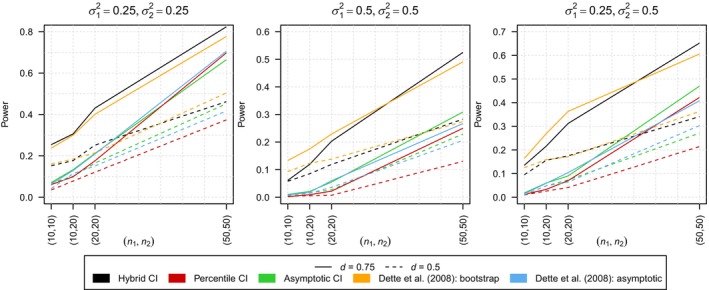
Comparison of the power of the CI‐based testing approaches to the testing approaches proposed by Dette et al. [10] with ε=1. The results are shown for two distances of the regression curves d∈{0.5, 0.75} and three different combinations of variances $\left(\sigma_{1}^{2},\sigma_{2}^{2}\right)\in \left\{\left(0.25, 0.25\right),\left(0.5, 0.5\right),\left(0.25, 0.5\right)\right\}$.

As the type I error rates are always smaller than the nominal level of α=0.05 for all three approaches (see Table S1 of the Supporting Information for exact values), that is, all testing approaches always hold the nominal level, the following analysis focuses on the power of the tests. Figure 1 shows the power for all three tests for all sub‐scenarios under the alternative as well as the corresponding power of the tests of Dette et al. [10] In each sub‐scenario we observe that the hybrid test has superior power compared to the other two CI‐based tests. In addition, one can observe that the power achieved by the hybrid test is quite similar to the one Dette et al. [10] observed for their bootstrap test and, therefore, is also superior to the power of their asymptotic test. The power of the test based on the percentile CI is considerably smaller which indicates that the test might be overly conservative in finite samples. The test based on the asymptotic CI leads to nearly the same results as the asymptotic test of Dette et al. [10] which is not surprising as it is directly derived from it. Consequentially, the lack of power that Dette et al. [10] observed for their asymptotic test in comparison to their bootstrap test is also present for the test based on the asymptotic CI.

In conclusion, the hybrid approach which provides numerical advantages compared to the bootstrap test of Dette et al. [10] and also leads to additional interpretability due to providing CIs, achieves nearly the same power as the bootstrap test while holding the nominal level.

### 4.2 Finite Sample Properties Under Model Uncertainty

We now investigate the finite sample properties under model uncertainty. Due to the clear superiority of the hybrid approach observed in Section 4.1, only the hybrid test is used for this analysis. We compare the performance of the test using model averaging to the one based on the correct specification of the models as well as under model misspecification. We use Bayesian model averaging with smooth BIC weights due to its theoretical advantages outlined in Section 3.2. However, as both candidate models have the same number of parameters, smooth BIC and smooth AIC weights are exactly equal as discussed in Section 2.2. For comparison, we additionally conduct the test based on model averaging with fixed equal weights, that is, $w_{l1}=w_{l2}=0.5,l=1,2$. The corresponding equivalence tests are conducted using Algorithm 1b.

#### 4.2.1 Comparison of an Emax With an Exponential Model

**FIGURE 2:**
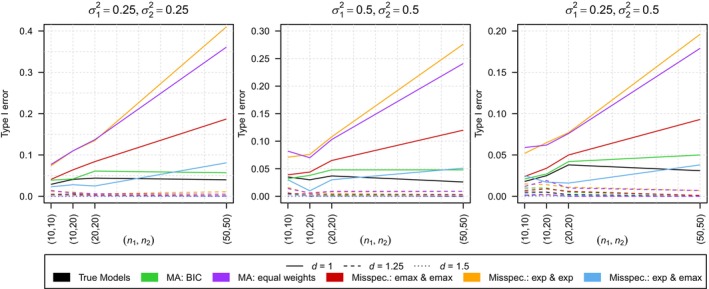
Comparison of the type I error rates of the test using the true model, the model averaging‐based test with smooth BIC weights (MA: BIC), the model averaging‐based test with equal weights (MA: equal weights) and the tests under model misspecification in scenario 1. The results are shown for ε=1, three distances of the regression curves d∈{1,1.25,1.5} and three different combinations of variances $\left(\sigma_{1}^{2},\sigma_{2}^{2}\right)\in \left\{\left(0.25, 0.25\right),\left(0.25, 0.5\right),\left(0.5, 0.5\right)\right\}$.

First, we again investigate the first simulation scenario introduced in Section 4.1 but now under model uncertainty where it is unclear if an emax or an exponential model applies for each of the groups implying $K_{1}=K_{2}=2$ and leading to one correct specification, as well as three misspecifications. Figure 2 shows the corresponding type I error rates for all sub‐scenarios under the null hypothesis, that is, for d∈{1, 1.25, 1.5} and $\left(\sigma_{1}^{2},\sigma_{2}^{2}\right)\in \left\{\left(0.25, 0.25\right),\left(0.25, 0.5\right),\left(0.5, 0.5\right)\right\}$ for the correct specification, the three misspecifications as well as under model averaging.

One can observe that falsely specifying the same model for both responses leads to highly inflated type I error rates which, in addition, even increase for increasing sample size. The highest type I errors are present if an exponential model is specified for both responses leading to type I error rates being as large as 0.410 which is observed for $\sigma_{1}^{2}=\sigma_{2}^{2}=0.25$ and $n_{1}=n_{2}=50$. If an emax model is specified for both responses, the type I error inflation is smaller but still present and reaches up to 0.187 which is observed for $\sigma_{1}^{2}=\sigma_{2}^{2}=0.25$ and $n_{1}=n_{2}=50$. The third misspecification under investigation is that specifying the models the wrong way round, that is, an exponential model for the first group and an emax model for the second one. In comparison to the other two misspecifications, this leads to less extreme results but type I error inflation is still observable. This results from the fact that using one convex (exponential) and one concave (emax) model usually leads to a larger maximal absolute deviation than using two convex or two concave models and, therefore, in general to fewer rejections of the null hypothesis.

Compared to these results, the type I errors resulting from model averaging with smooth BIC weights are closer to the nominal level of the test. However, for two out of the 36 investigated sub‐scenarios ($\sigma_{1}^{2}=\sigma_{2}^{2}=0.25$ and $n_{1}=n_{2}=20$ as well as $n_{1}=n_{2}=50$) the type I errors still exceeds the nominal level but to a much lesser extent compared to model misspecification, reaching a maximum of 0.061. In contrast, using model averaging with equal weights leads to a high type I error inflation similar to the one observed under model misspecification. As expected, when using the true underlying model, the test holds the nominal level of α=0.05. Comparison of the power of the tests is not meaningful as some of them are not holding the nominal level. However, the estimated power is shown in Table S2 of the Supporting Information.

#### 4.2.2 Comparison of Two Shifted Emax Models

We continue by investigating the fine sample properties for the case of two shifted emax models, that is, model (3) now applies for both groups, where $\theta_{1}=\left(\beta_{10},\beta_{11},\beta_{12}\right)=\left(\beta_{10},5,1\right)$ and $\theta_{2}=\left(\beta_{20},\beta_{21},\beta_{22}\right)=\left(0,5,1\right)$, which implies $d=\beta_{10}$. The levels of d under investigation are 1, 0.75, 0.5, 0.25, 0.1 and 0. The test is conducted for ε=0.5 such that the first three deviations are under the null hypothesis and, therefore, used to investigate the type I error rates. The latter three deviations are under the alternative and used to estimate the power of the tests.

**FIGURE 3:**
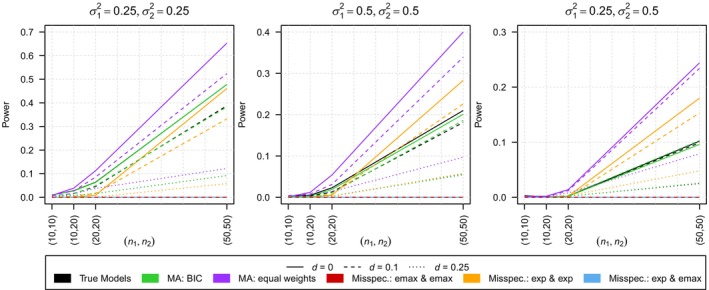
Comparison of the power of the test using the true model, the model averaging‐based test with smooth BIC weights (MA: BIC), the model averaging‐based test with equal weights (MA: equal weights) and the tests under model misspecification in scenario 2. The results are shown for ε=0.5, three distances of the regression curves d∈{1, 1.25, 1.5} and three different combinations of variances $\left(\sigma_{1}^{2},\sigma_{2}^{2}\right)\in \left\{\left(0.25, 0.25\right),\left(0.25, 0.5\right),\left(0.5, 0.5\right)\right\}$.

We only observe few type I error rates which are non‐zero and these are still much smaller than the nominal level of α=0.05, reaching a maximum of only 0.003 (all values can be found in Table S3 of the Supporting Information). Hence, the analysis focuses on the power of the tests which is shown in Figure 3.

One can observe falsely specifying one of the models to be an exponential model leads to the power being constantly equal to zero even for sub‐scenarios which are quite far under the alternative. For misspecification in terms of using an exponential model for both responses, the power loss is not that extensive but still occurs for smaller sample sizes, which is especially visible for $\sigma_{1}^{2}=\sigma_{2}^{2}=0.25$ due to the estimation uncertainty being the smallest. In contrast, model averaging with smooth BIC weights results in nearly the same power as using the true model. This also leads to the fact that in some cases the black line is even hardly visual as it is nearly perfectly overlapped by the green one. The use of model averaging with equal weights leads to high power, even exceeding the power obtained by using the true models. Therefore, this high number of rejections of the null hypothesis may not be caused by the model fitting the data well, but by forcing the data into a (partly) wrong model, similar to what can be observed in some scenarios when using two exponential models.

#### 4.2.3 Comparison of Two Shifted Exponential Models

The third simulation scenario is given by two shifted exponential models, that is, model (4) now applies for both groups, where $\theta_{1}=\left(\beta_{10},\beta_{11},\beta_{12}\right)=\left(\beta_{10},2.2, 8\right)$ and $\theta_{2}=\left(\beta_{20},\beta_{21},\beta_{22}\right)=\left(0, 2.2, 8\right)$ which implies $d=\beta_{10}$, resulting in the same values for d as in Section 4.2.2. The test is conducted for ε=0.5 such that the first three deviations are under the null hypothesis and, therefore, used to investigate the type I error rates. The latter three deviations are under the alternative and used to estimate the power of the tests.

**FIGURE 4:**
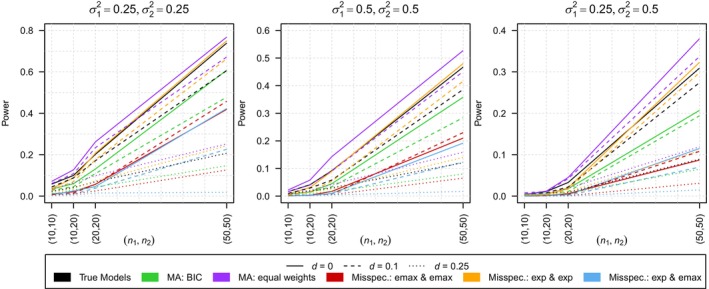
Comparison of the power of the test using the true model, the model averaging‐based test with smooth BIC weights (MA: BIC), the model averaging‐based test with equal weights (MA: equal weights) and the tests under model misspecification in scenario 3. The results are shown for ε=0.5, three distances of the regression curves d∈{1, 1.25, 1.5} and three different combinations of variances $\left(\sigma_{1}^{2},\sigma_{2}^{2}\right)\in \left\{\left(0.25, 0.25\right),\left(0.25, 0.5\right),\left(0.5, 0.5\right)\right\}$.

As previously observed in Section 4.2.2 only few type I error rates are non‐zero and these exceptions are still much smaller than the nominal level of α=0.05, reaching a maximum of only 0.009 (all values can be found in Table S4 of the Supporting Information). Hence, the analysis focuses on the power of the tests which is shown in Figure 4. The loss of power resulting from model misspecification is not as large as in Section 4.2.2 but still present. Especially if one of the models is falsely specified to be an emax model but the other one specified correctly, we observe a notable loss of power not only compared to using the true model but also compared to using model averaging. Moreover, this effect is increasing with increasing sample size. In contrast to Section 4.2.2, the power resulting from using model averaging with smooth BIC weights is notably smaller than the one observed when using the true model. However, compared to two out of the three misspecifications, the loss in power is extensively reduced. Similar to Section 4.2.2, using model averaging with equal weights leads to a high power even exceeding the power which results from using the true models. As in Section 4.2.2, this might be caused by forcing the data into a (partly) wrong model rather than by the model fitting the data well. In conclusion, if the models are misspecified we observe either type I error inflation or a lack of power, both often of substantial extend, in all three scenarios. Using model averaging with smooth BIC weights considerably reduces these problems, often leading to similar results as knowing and using the true underlying model.

## 5 Case Study

We illustrate the proposed methodology through a case study analyzing the equivalence of time‐response curves (also known as exposure duration‐response curves) using data which was originally published by Ghallab et al. [47] The study aims to investigate dietary effects onto the gene expression. The dataset consists of two groups of mice which were fed with two different diets and then sacrificed at different time points. The first one is a high‐fat or “Western” diet (WD) while the other one is a standard diet (SD). As no data has been collected in the first 3 weeks, they are not included into our analysis. Consequentially, the beginning of the study (t=0) resembles week 3 of the actual experiment. Data is then observed at t=0,3,9, 15, 21, 27, 33, 39, and 45 for the Western diet and at t=0,3,27, 33, 39, and 45 for the standard diet with sample sizes 5,5,5,5,5,5,5,4,8 and 7,5,5,7,3,5, respectively. For each group, the gene expression of 20 733 genes is measured in terms of gene counts. For our analysis, we focus on the 1000 genes Ghallab et al. [47] classified as especially interesting due to high activity. Although gene expression is measured as count data, it is treated as continuous due to the very high number of counts. The raw count data is preprocessed in terms of the gene count normalization conducted by Ghallab et al. [47] and subsequent log_2_‐transformation of the normalized counts as suggested by Duda et al. [42, 48]

Using this data, we aim to investigate the equivalence of the time‐gene expressions curves between the two diets at a 5% significance level. From Ghallab et al. [47] it is known that there are quite large differences between the diets, such that for the majority of the genes we expect not to conclude equivalence. However, precisely for this reason it is of interest for which genes equivalence can be concluded nevertheless. As we are interested in the results for each gene separately and are not aiming for a global conclusion, we do not adjust for multiple testing.

As time‐response studies are relatively rare, no specific time‐response models have been developed. Hence, dose–response models are deployed for time‐response relationsas well. Methodological review studies [49] do also not distinguish between dose–response and time‐response studies. In addition, it seems intuitive that the effects of the high‐fat diet accumulate with increasing time of consumption in a similar manner as the effects in dose response‐studies accumulate with increasing dose.

As outlined by Ghallab et al. [47] the dose–response relations vary across genes such that there is no single model which fits to all of them and, hence, model uncertainty is present. In addition, the models cannot be chosen manually due to the high number of genes. We introduce model averaging using BIC‐based weights and the equivalence tests are performed using hybrid CI, that is, by conducting Algorithm 1b. We deploy the set of candidate models suggested by Duda et al. [42], that is, a linear model (1), a quadratic model (2), an emax model (3), an exponential model (4), a sigmoid emax model (5), and a beta model (6). This set of candidate models can capture quite diverse effects, as it includes linear and nonlinear, increasing and decreasing, monotone and non‐monotone as well as convex, concave and sigmoid curves.

The ranges of the response variables, that is, the ranges of log_2_ (normalized counts), are not comparable across different genes. Hence, different equivalence thresholds are needed for each of the genes. As such thresholds can not be chosen manually due to the high number of genes, we determine the thresholds as a percentile of the range of the response variable. For a gene g∈{1,…,1000}, that is

$$\varepsilon_{g}=\tilde{\varepsilon }\left(\max_{l,i}\left({\hat{y}}_{\mathrm{gli}}\right)-\min_{l,i}\left({\hat{y}}_{\mathrm{gli}}\right)\right)$$

where $\tilde{\varepsilon }\in \left(0,1\right)$ is the corresponding percentile and $\tilde{\varepsilon }=0.2$ or 0.25 would be typical choices. Alternatively, one can proceed the other way around, calculate

$${\tilde{u}}_{g}=\frac{u_{g}}{\max_{l,i}\left({\hat{y}}_{\mathrm{gli}}\right)-\min_{l,i}\left({\hat{y}}_{\mathrm{gli}}\right)}$$

and directly compare ${\tilde{u}}_{g}$ to $\tilde{\varepsilon }$, that is, the decision rules $\varepsilon_{g}>u_{g}$ and $\tilde{\varepsilon }>{\tilde{u}}_{g}$ are equivalent.

**FIGURE 5:**
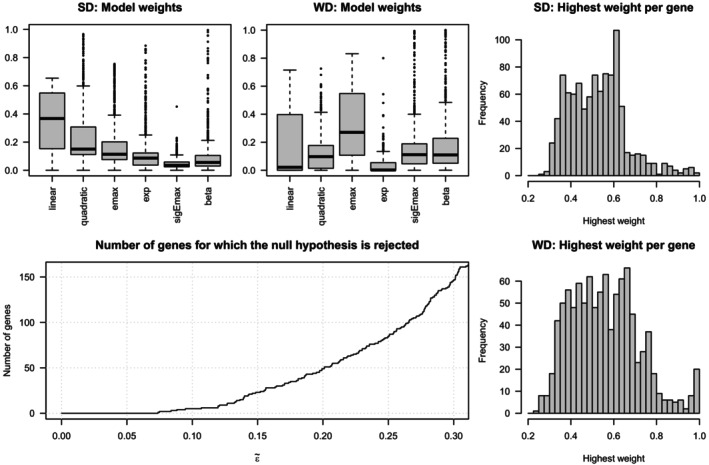
Subfigures (a) and (b) show boxplots of the model weights for each of the two diets. Subfigures (c) and (d) show histograms of the highest model weight per gene for both genes. Subfigure (e) shows the number of genes for which equivalence between the time‐gene expression curves of the two diets can be concluded in dependence of the equivalence threshold $\tilde{\varepsilon }$.

Figure 5a,b show boxplots of the model weights for both diets. It can be observed that less complex models, the linear and quadratic model, have higher weights for the standard diet compared to the Western diet. In contrast, for the two most complex models, the beta and sigEmax model, the opposite can be observed: they have higher weights for the Western diet compared to the standard diet.

In addition, Figure 5c,d show histograms of the highest model weight per gene. It can be observed that for the Western diet the model weights tend to be larger compared to the standard diet. In addition, for the Western diet there are notably many genes for which the highest model weight is very close to one, that is, the averaged model consists nearly fully of only one of the candidate models.

Figure 5e shows the number of genes for which equivalence of the time‐gene expression curves of both diets can be concluded, that is, the number of genes for which $H_{0}$ can be rejected depending on the choice of $\tilde{\varepsilon }$. For very small choices of $\tilde{\varepsilon }$ (e.g., 0.05, 0.06, or 0.07) equivalence cannot be concluded for any gene and for $\tilde{\varepsilon }=0.1$ only five genes would be assessed as equivalent. For more typical choices of $\tilde{\varepsilon }$ being 0.2, 0.25, or 0.3, equivalence could be concluded for 50, 85, and 147 genes, respectively. With further increasing $\tilde{\varepsilon }$ the number of rejections also further increases and approaches 1000. However, this is not shown for $\tilde{\varepsilon }>0.3$ as performing an equivalence test with a threshold larger than 30% of the range of the response variable might not have practical relevance.

**FIGURE 6:**
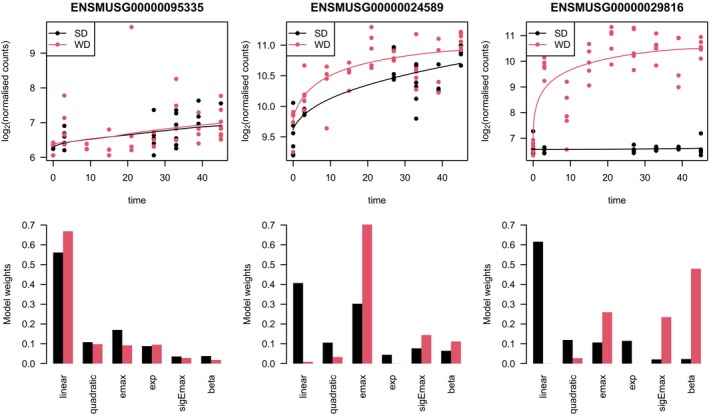
Results for three exemplary genes. The first row of figures shows the data for both diets as well as the fitted models. The second row of figures shows the corresponding model weights.

Figure 6 shows the results for three exemplary genes. For ENSMUSG00000095335 it can be observed that both time‐response curves are extremely close to each other and that the maximum absolute deviation of the curves is quite small. This leads to $\tilde{u}\approx 0.084$. Regarding the model weights it can be observed that both time‐response curves consist essentially of the same models. For ENSMUSG00000024589 we observe that both time‐response curves have a similar shape both being emax‐like, although their model weights are not as similar as before. However, their distance is larger than for ENSMUSG00000095335 which leads to $\tilde{u}\approx 0.303$. Hence, the curves are not equivalent for typical choices of $\tilde{\varepsilon }$ being, for example, 0.2 or 0.3 but only for extremely liberal choices of $\tilde{\varepsilon }$, for example, for $\tilde{\varepsilon }=0.35$. For the last example ENSMUSG00000029816 we observe that the two curves are completely different with regard to both, shape and location. For the standard diet an almost constant curve is present while for the Western diet a typical emax shape is observable. This is also reflected by the model weights where models which have high weights for one curve, have small ones for the other one and vise versa, the only exempt to this is the emax model which has a medium large weight for both of the groups. Due to the large maximum absolute deviation between the curves given by $\hat{d}\approx 3.891$, similarity cannot be concluded for any reasonable equivalence threshold.

## 6 Conclusion

In this paper, we introduced a new approach for model‐based equivalence testing which can also be applied in the presence of model uncertainty — a problem which is usually faced in practical applications. Our approach is based on a flexible model averaging method which relies on information criteria and a testing procedure which makes use of the duality of tests and confidence intervals rather than simulating the distribution under the null hypothesis, providing a numerically stable procedure. Due to the advantages of theoretical validity based on asymptotic theory, we chose to use the BIC as the information criterion. Moreover, our approach leads to additional interpretability due to the provided confidence intervals while retaining the asymptotic validity and a similar performance in finite samples as the bootstrap based test proposed by Dette et al. [10].

Precisely, we investigated the finite sample properties of the proposed method by reanalysing the simulation study of Dette et al. [10] and observed similar results for the CI‐based test compared to their test. In the presence of model uncertainty, model misspecification frequently led to either type I error inflation or a lack of power, both often of substantial extend. In contrast, our approach considerably reduced these problems and in many cases even achieved similar results as knowing and using the true underlying model. In direct comparison, a simpler model averaging method, that is, using fixed equal weights, was not able to prevent high type I error inflation. Therefore, we strongly recommend to use information criteria‐based model averaging. The presented case study outlines the practical usefulness of the proposed method based on a large data application where choosing the models manually would be time‐consuming and could easily lead to many model misspecifications. Hence, introducing model averaging here is essential to test for the equivalence of time‐gene expression curves for such large numbers of genes, typically occurring in practice.

Future possible research includes extending the presented method for other model averaging techniques, for example, cross validation‐based model averaging. In addition, transferring this approach to other model classes (e.g., survival models) as well as to multidimensional responses, that is, multiple endpoints, merits further exploration.

## Conflicts of Interest

The authors declare no conflicts of interest.

## Supporting information


**Data S1**. Supporting information.

File S1.

## Data Availability

Software in the form of R code available at https://github.com/Niklas191/equivalence_tests_with_model_averaging.git. The case study data set is publicly available at the SRA database with reference number PRJNA953810.
