# Supplementary material for: Overcoming Model Uncertainty — How Equivalence Tests Can Benefit From Model Averaging
Source: Stat Med. 2025 Mar 19;44(6):e10309. doi: 10.1002/sim.10309 (PMC11923417; doi:10.1002/sim.10309)
Supplement: Supplementary file 1 — Data S1. Supporting information. [file SIM-44-0-s001.pdf]

Overcoming model uncertainty – how equivalence tests can benefit from model averaging

Supporting information

Niklas Hagemann[1] and Kathrin Möllenhoff[1]

[1] Institute of Medical Statistics and Computational Biology (IMSB),

Faculty of Medicine, University of Cologne, Germany

October 21, 2024

S 1: Comparison of the type I error rates of the CI-based testing approaches to the testing approaches proposed by Dette et al. (2018) with $\varepsilon = 1$. The results are shown for two distances of the regression curves $d \in \{0.5, 0.75\}$ and three different combinations of variances $(\sigma_1^2, \sigma_2^2) \in \{(0.25, 0.25), (0.5, 0.5), (0.25, 0.5)\}$.

β_{20}	d	σ_1^2	σ_2^2	n_1	n_2	hybrid CI	percentile CI	asymptotic CI	Dette et al. (2018) bootstrap	Dette et al. (2018) asymptotic
0.25	1.50	0.25	0.25	10	10	0.000	0.000	0.000	0.001	0.000
0.25	1.50	0.25	0.25	10	20	0.000	0.000	0.000	0.000	0.000
0.25	1.50	0.25	0.25	20	20	0.000	0.000	0.000	0.000	0.000
0.25	1.50	0.25	0.25	50	50	0.000	0.000	0.000	0.000	0.000
0.25	1.50	0.25	0.50	10	10	0.000	0.000	0.000	0.000	0.000
0.25	1.50	0.25	0.50	10	20	0.000	0.000	0.000	0.000	0.000
0.25	1.50	0.25	0.50	20	20	0.000	0.000	0.000	0.000	0.000
0.25	1.50	0.25	0.50	50	50	0.000	0.000	0.000	0.000	0.000
0.25	1.50	0.50	0.50	10	10	0.001	0.000	0.000	0.001	0.000
0.25	1.50	0.50	0.50	10	20	0.001	0.000	0.000	0.002	0.000
0.25	1.50	0.50	0.50	20	20	0.000	0.000	0.000	0.000	0.000
0.25	1.50	0.50	0.50	50	50	0.000	0.000	0.000	0.000	0.000
0.50	1.25	0.25	0.25	10	10	0.004	0.000	0.000	0.005	0.001
0.50	1.25	0.25	0.25	10	20	0.005	0.004	0.001	0.004	0.000
0.50	1.25	0.25	0.25	20	20	0.001	0.000	0.000	0.001	0.000
0.50	1.25	0.25	0.25	50	50	0.000	0.000	0.000	0.000	0.000
0.50	1.25	0.25	0.50	10	10	0.006	0.001	0.002	0.006	0.000
0.50	1.25	0.25	0.50	10	20	0.000	0.000	0.002	0.005	0.001
0.50	1.25	0.25	0.50	20	20	0.001	0.000	0.000	0.000	0.001
0.50	1.25	0.25	0.50	50	50	0.000	0.000	0.000	0.000	0.000
0.50	1.25	0.50	0.50	10	10	0.005	0.001	0.000	0.011	0.001
0.50	1.25	0.50	0.50	10	20	0.005	0.000	0.001	0.013	0.005
0.50	1.25	0.50	0.50	20	20	0.002	0.000	0.000	0.004	0.000
0.50	1.25	0.50	0.50	50	50	0.000	0.000	0.000	0.000	0.000
0.75	1.00	0.25	0.25	10	10	0.029	0.007	0.005	0.045	0.012
0.75	1.00	0.25	0.25	10	20	0.041	0.008	0.015	0.045	0.019
0.75	1.00	0.25	0.25	20	20	0.044	0.016	0.015	0.034	0.011
0.75	1.00	0.25	0.25	50	50	0.040	0.016	0.027	0.051	0.016
0.75	1.00	0.25	0.50	10	10	0.035	0.004	0.005	0.036	0.003
0.75	1.00	0.25	0.50	10	20	0.030	0.005	0.009	0.028	0.009
0.75	1.00	0.25	0.50	20	20	0.037	0.009	0.010	0.048	0.009
0.75	1.00	0.25	0.50	50	50	0.026	0.008	0.014	0.058	0.012
0.75	1.00	0.50	0.50	10	10	0.018	0.001	0.002	0.037	0.005
0.75	1.00	0.50	0.50	10	20	0.025	0.002	0.001	0.046	0.006
0.75	1.00	0.50	0.50	20	20	0.038	0.003	0.005	0.038	0.036
0.75	1.00	0.50	0.50	50	50	0.031	0.010	0.010	0.059	0.015

S 2: Comparison of the power of the test using the true model, the model averaging-based tests and the tests under model misspecification in scenario 1. The results are shown for $\varepsilon = 1$, three distances of the regression curves $d \in \{1, 1.25, 1.5\}$ and three different combinations of variances $(\sigma_1^2, \sigma_2^2) \in \{(0.25, 0.25), (0.25, 0.5), (0.5, 0.5)\}$.

β_{20}	d	σ_1^2	σ_2^2	n_1	n_2	true model	MA: BIC	MA: EW	emax & emax	exp & exp	exp & emax
1.0	0.75	0.25	0.25	10	10	0.152	0.173	0.296	0.190	0.293	0.133
1.0	0.75	0.25	0.25	10	20	0.177	0.213	0.385	0.239	0.383	0.173
1.0	0.75	0.25	0.25	20	20	0.252	0.283	0.539	0.359	0.553	0.246
1.0	0.75	0.25	0.25	50	50	0.462	0.508	0.937	0.693	0.932	0.659
1.0	0.75	0.25	0.50	10	10	0.095	0.107	0.204	0.112	0.193	0.096
1.0	0.75	0.25	0.50	10	20	0.156	0.168	0.305	0.190	0.284	0.135
1.0	0.75	0.25	0.50	20	20	0.175	0.215	0.386	0.260	0.379	0.152
1.0	0.75	0.25	0.50	50	50	0.341	0.408	0.800	0.593	0.784	0.462
1.0	0.75	0.50	0.50	10	10	0.058	0.071	0.143	0.078	0.129	0.071
1.0	0.75	0.50	0.50	10	20	0.085	0.099	0.204	0.105	0.204	0.107
1.0	0.75	0.50	0.50	20	20	0.119	0.143	0.283	0.157	0.290	0.127
1.0	0.75	0.50	0.50	50	50	0.283	0.338	0.662	0.445	0.689	0.336
1.5	0.50	0.25	0.25	10	10	0.254	0.316	0.575	0.363	0.589	0.576
1.5	0.50	0.25	0.25	10	20	0.306	0.387	0.658	0.435	0.700	0.727
1.5	0.50	0.25	0.25	20	20	0.431	0.506	0.850	0.597	0.889	0.917
1.5	0.50	0.25	0.25	50	50	0.824	0.847	0.995	0.930	0.997	0.999
1.5	0.50	0.25	0.50	10	10	0.135	0.177	0.371	0.214	0.345	0.323
1.5	0.50	0.25	0.50	10	20	0.216	0.293	0.530	0.340	0.536	0.544
1.5	0.50	0.25	0.50	20	20	0.312	0.375	0.699	0.462	0.724	0.725
1.5	0.50	0.25	0.50	50	50	0.652	0.690	0.974	0.839	0.974	0.994
1.5	0.50	0.50	0.50	10	10	0.061	0.097	0.226	0.108	0.214	0.183
1.5	0.50	0.50	0.50	10	20	0.119	0.177	0.327	0.201	0.349	0.332
1.5	0.50	0.50	0.50	20	20	0.203	0.289	0.536	0.326	0.571	0.551
1.5	0.50	0.50	0.50	50	50	0.525	0.552	0.912	0.698	0.930	0.961

S 3: Comparison of the type I error rates of the test using the true model, the model averaging-based tests and the tests under model misspecification in scenario 2. The results are shown for $\varepsilon = 0.5$, three distances of the regression curves $d \in \{1, 1.25, 1.5\}$ and three different combinations of variances $(\sigma_1^2, \sigma_2^2) \in \{(0.25, 0.25), (0.25, 0.5), (0.5, 0.5)\}$.

$\beta_{10} = d$	σ_1^2	σ_2^2	n_1	n_2	true model	MA: BIC	MA: EW	emax & exp	exp & exp	exp & emax
0.50	0.25	0.25	10	10	0.001	0.000	0.000	0.000	0.000	0.000
0.50	0.25	0.25	10	20	0.001	0.001	0.000	0.000	0.000	0.000
0.50	0.25	0.25	20	20	0.003	0.003	0.005	0.000	0.000	0.000
0.50	0.25	0.25	50	50	0.001	0.001	0.002	0.000	0.000	0.000
0.50	0.25	0.50	10	10	0.001	0.000	0.000	0.000	0.000	0.000
0.50	0.25	0.50	10	20	0.001	0.000	0.005	0.000	0.000	0.000
0.50	0.25	0.50	20	20	0.001	0.001	0.003	0.000	0.000	0.000
0.50	0.25	0.50	50	50	0.001	0.001	0.001	0.000	0.000	0.000
0.50	0.50	0.50	10	10	0.000	0.000	0.000	0.000	0.000	0.000
0.50	0.50	0.50	10	20	0.000	0.000	0.000	0.000	0.000	0.000
0.50	0.50	0.50	20	20	0.000	0.000	0.002	0.000	0.000	0.000
0.50	0.50	0.50	50	50	0.000	0.000	0.002	0.000	0.000	0.000
0.75	0.25	0.25	10	10	0.000	0.000	0.000	0.000	0.000	0.000
0.75	0.25	0.25	10	20	0.000	0.000	0.000	0.000	0.000	0.000
0.75	0.25	0.25	20	20	0.000	0.000	0.000	0.000	0.000	0.000
0.75	0.25	0.25	50	50	0.000	0.000	0.000	0.000	0.000	0.000
0.75	0.25	0.50	10	10	0.000	0.000	0.000	0.000	0.000	0.000
0.75	0.25	0.50	10	20	0.000	0.000	0.000	0.000	0.000	0.000
0.75	0.25	0.50	20	20	0.000	0.000	0.000	0.000	0.000	0.000
0.75	0.25	0.50	50	50	0.000	0.000	0.000	0.000	0.000	0.000
0.75	0.50	0.50	10	10	0.000	0.000	0.000	0.000	0.000	0.000
0.75	0.50	0.50	10	20	0.000	0.000	0.000	0.000	0.000	0.000
0.75	0.50	0.50	20	20	0.000	0.000	0.000	0.000	0.000	0.000
0.75	0.50	0.50	50	50	0.000	0.000	0.000	0.000	0.000	0.000
1.00	0.25	0.25	10	10	0.000	0.000	0.000	0.000	0.000	0.000
1.00	0.25	0.25	10	20	0.000	0.000	0.000	0.000	0.000	0.000
1.00	0.25	0.25	20	20	0.000	0.000	0.000	0.000	0.000	0.000
1.00	0.25	0.25	50	50	0.000	0.000	0.000	0.000	0.000	0.000
1.00	0.25	0.50	10	10	0.000	0.000	0.000	0.000	0.000	0.000
1.00	0.25	0.50	10	20	0.000	0.000	0.000	0.000	0.000	0.000
1.00	0.25	0.50	20	20	0.000	0.000	0.000	0.000	0.000	0.000
1.00	0.25	0.50	50	50	0.000	0.000	0.000	0.000	0.000	0.000
1.00	0.50	0.50	10	10	0.000	0.000	0.001	0.000	0.000	0.000
1.00	0.50	0.50	10	20	0.000	0.000	0.000	0.000	0.000	0.000
1.00	0.50	0.50	20	20	0.000	0.000	0.000	0.000	0.000	0.000
1.00	0.50	0.50	50	50	0.000	0.000	0.000	0.000	0.000	0.000

S 4: Comparison of the type I error rates of the test using the true model, the model averaging-based tests and the tests under model misspecification in scenario 3. The results are shown for $\varepsilon = 0.5$, three distances of the regression curves $d \in \{1, 1.25, 1.5\}$ and three different combinations of variances $(\sigma_1^2, \sigma_2^2) \in \{(0.25, 0.25), (0.25, 0.5), (0.5, 0.5)\}$.

$\beta_{10} = d$	σ_1^2	σ_2^2	n_1	n_2	true model	MA: BIC	MA: EW	emax & exp	emax & emax	exp & emax
0.50	0.25	0.25	10	10	0.005	0.002	0.007	0.000	0.000	0.000
0.50	0.25	0.25	10	20	0.005	0.002	0.006	0.001	0.002	0.000
0.50	0.25	0.25	20	20	0.005	0.006	0.010	0.002	0.006	0.000
0.50	0.25	0.25	50	50	0.001	0.001	0.001	0.000	0.002	0.000
0.50	0.25	0.50	10	10	0.002	0.000	0.003	0.002	0.001	0.000
0.50	0.25	0.50	10	20	0.009	0.003	0.011	0.001	0.003	0.000
0.50	0.25	0.50	20	20	0.002	0.001	0.006	0.000	0.003	0.000
0.50	0.25	0.50	50	50	0.000	0.000	0.001	0.001	0.001	0.000
0.50	0.50	0.50	10	10	0.002	0.001	0.002	0.000	0.001	0.001
0.50	0.50	0.50	10	20	0.001	0.001	0.002	0.000	0.000	0.000
0.50	0.50	0.50	20	20	0.003	0.002	0.004	0.000	0.001	0.000
0.50	0.50	0.50	50	50	0.003	0.001	0.004	0.002	0.003	0.000
0.75	0.25	0.25	10	10	0.000	0.000	0.000	0.000	0.000	0.000
0.75	0.25	0.25	10	20	0.000	0.000	0.001	0.000	0.001	0.000
0.75	0.25	0.25	20	20	0.000	0.000	0.000	0.000	0.000	0.000
0.75	0.25	0.25	50	50	0.000	0.000	0.000	0.000	0.000	0.000
0.75	0.25	0.50	10	10	0.000	0.000	0.000	0.000	0.000	0.000
0.75	0.25	0.50	10	20	0.000	0.000	0.000	0.000	0.000	0.000
0.75	0.25	0.50	20	20	0.000	0.000	0.000	0.000	0.000	0.000
0.75	0.25	0.50	50	50	0.000	0.000	0.000	0.000	0.000	0.000
0.75	0.50	0.50	10	10	0.000	0.000	0.000	0.000	0.000	0.000
0.75	0.50	0.50	10	20	0.001	0.000	0.001	0.000	0.000	0.000
0.75	0.50	0.50	20	20	0.001	0.000	0.001	0.000	0.000	0.000
0.75	0.50	0.50	50	50	0.000	0.000	0.000	0.000	0.000	0.000
1.00	0.25	0.25	10	10	0.000	0.000	0.000	0.000	0.000	0.000
1.00	0.25	0.25	10	20	0.000	0.000	0.000	0.000	0.000	0.000
1.00	0.25	0.25	20	20	0.000	0.000	0.000	0.000	0.000	0.000
1.00	0.25	0.25	50	50	0.000	0.000	0.000	0.000	0.000	0.000
1.00	0.25	0.50	10	10	0.000	0.000	0.000	0.000	0.000	0.000
1.00	0.25	0.50	10	20	0.000	0.000	0.000	0.000	0.000	0.000
1.00	0.25	0.50	20	20	0.000	0.000	0.000	0.000	0.000	0.000
1.00	0.25	0.50	50	50	0.000	0.000	0.000	0.000	0.000	0.000
1.00	0.50	0.50	10	10	0.000	0.000	0.001	0.000	0.001	0.000
1.00	0.50	0.50	10	20	0.000	0.000	0.000	0.000	0.000	0.000
1.00	0.50	0.50	20	20	0.000	0.000	0.000	0.000	0.000	0.000
1.00	0.50	0.50	50	50	0.000	0.000	0.000	0.000	0.000	0.000
